# A multilevel study of patient-centered care perceptions in mental health teams

**DOI:** 10.1186/s12913-020-06054-z

**Published:** 2021-01-07

**Authors:** François Durand, Marie-Josée Fleury

**Affiliations:** 1 Montfort Research Chair in Organization of Health Services, Telfer School of Management, University of Ottawa, 55 Laurier Avenue East, Ottawa, On K1N 6N5 Canada; 2 Douglas Mental Health University Institute, McGill University, 6875 LaSalle Blvd, Montreal, Qc H4H 1R3 Canada

**Keywords:** Patient-centered care, Teamwork, Collaboration, Adaptation, Attitudes, Beliefs

## Abstract

**Background:**

The successful combination of interprofessional collaboration in multidisciplinary teams with patient-centered care is necessary when it comes to delivering complex mental health services. Yet collaboration is challenging and patient-centered care is intricate to manage. This study examines correlates of patient-centered care such as team adaptivity and proactivity, collaboration, belief in interprofessional collaboration and informational role self-efficacy in multidisciplinary mental health teams.

**Method:**

A cross-sectional multilevel survey design was used, based on self-administered bilingual validated questionnaires. Participants (*N*=314) were mental health professionals and managers working in public primary care or specialized mental health services, in inpatient or outpatient settings.

**Results:**

This study showed that belief in interprofessional collaboration’s relationship with patient-centered perceptions is increased in teams with high collaboration. Collaboration is also found as a mediator, representing a process by which team adaptive and proactive behaviors are transformed into positive patient-centered perceptions.

**Conclusions:**

Our results were in line with recent studies on team processes establishing that collaboration is a key component in multilevel examinations of predictors of patient-centered care. In terms of practice, our study showed that multidisciplinary teams should know that working hard on collaboration is an answer to the complexity of patient-centered care. Collaboration is related to the teams’ ability to respond to its challenges. It is also related to individuals’ beliefs central to the delivery of interprofessional care.

## Background

Interprofesional collaboration occurs in a team context [1] and is “a type of professional work which involves different health and social care professions who regularly come together to solve problems or provide services” [2]. Patient-centred care is based on the unique needs of the patient and on the interpersonal relationship with care providers that enables these needs to be understood [3]. Some consider interprofessional collaboration as inherently patient-centred [4]. Furthermore, the successful combination of interprofessional collaboration in multidisciplinary teams with patient-centered care is ncessary when it comes to delivering complex mental health services. Indeed, biopsychosocial roots of mental health problems impact multiple aspects of patients’ lives [5]. A plurality of views from the part of health care professionals are essential for providing all-inclusive, rounded services that meet patients’ complex needs [6, 7].

Interprofessional collaboration in multidisciplinary teams is effective in mental health settings. For example, it is found to improve patient health status and treatment compliance, reduce suicides and clinical errors, boost professionals’ satisfaction and motivation, lower admission rates and shorten stays [8–11]. Multidisciplinary teams however face key challenges in implementing interprofessional collaboration such as barriers caused by different professional cultures [12], divergent values [13], and lack of recognition of each others’ roles [14]. Patient-centered care is also intricate to manage [15] as teams need to consider more factors in delivering services. The delivery and implementation of optimal patient-centered care within mental health settings is a real challenge [16]. Collaborative relationships in mental health care teams are difficult to implement, require time, work and supportive structures [17] to address barriers including power differences, time constraints, medical dominance, communication challenges and lack of resources [18–20]. In general people agree: multidisciplinary teams work better for complex patients’ needs but they are challenging to manage for team members [17]. This study addresses some of these challenges by investigating the central role played by collaboration.

The objective of this study is to examine the role of potential correlates of patient-centered care perceptions in multidisciplinary mental health teams. The complexity of multidisciplinary teams calls for a multilevel approach where some variables are at the individual level while others are at the team level. This study will thus examine the role played by two important individual-level attitudes: belief in the benefits of interprofessional collaboration and informational role self-efficacy. This study looks at how collaboration at the team level potentially relates to these variables. Finally, this study examines the role collaboration plays in linking team work role behaviors to team-level patient-centered care perceptions.

### Individual- and team-level perspective

Team interactions in general and collaboration in particular are team processes, that is, they are “acts that convert inputs to outcomes through cognitive, verbal, and behavioral activities directed toward organizing taskwork to achieve collective goals” [21]. Specifically, collaboration is the interplay of four processes: teamwork communication, synchronicity, explicit coordination, and implicit coordination [22]. Communication involves effective information exchange. Synchronicity encompasses working with others on time and in time. Explicit coordination involves overt exchanges on role and task assignments and implicit coordination consist of anticipation of others’ needs without resorting to explicit coordination. Processes and the interactions they foster act as a social context impacting team members’ behaviors and attitudes [23, 24].

**Fig. 1:**
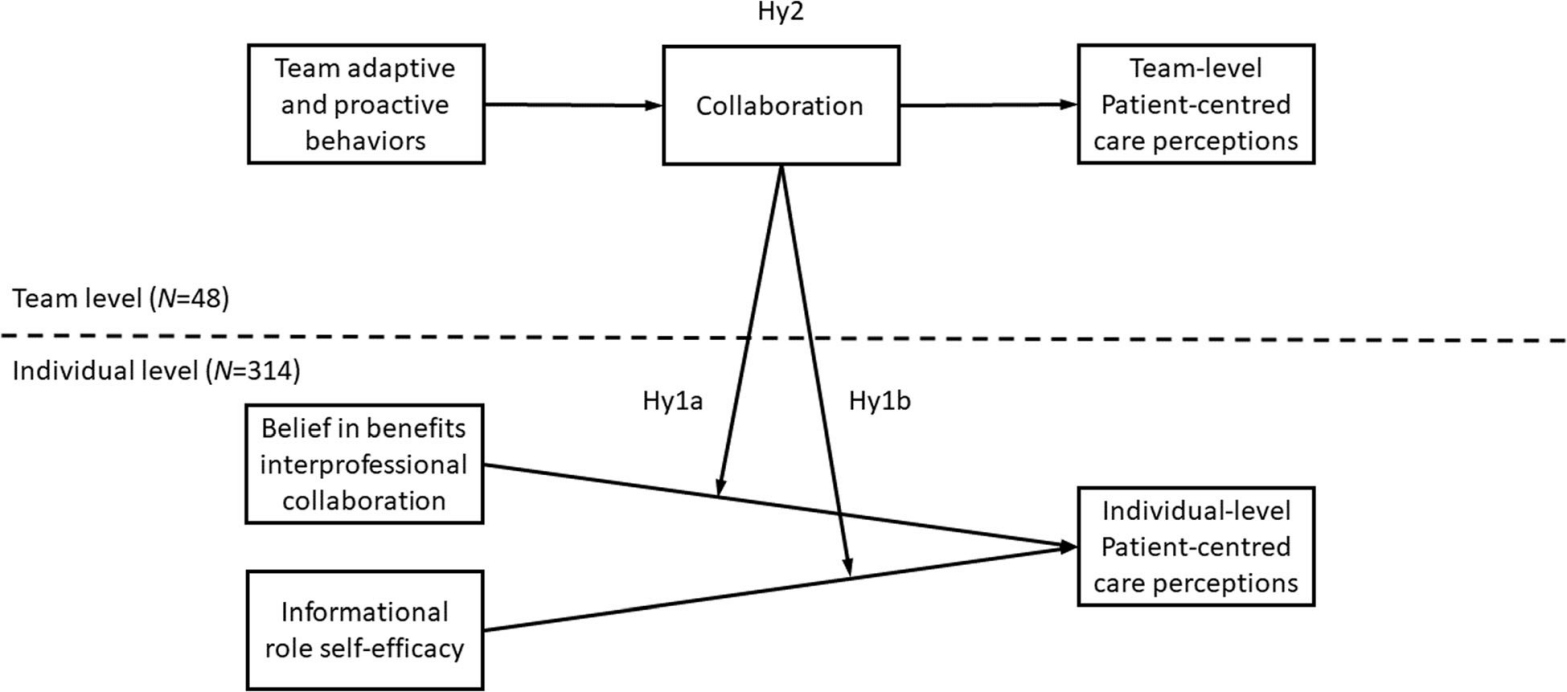
Visual representation of the multilevel hypotheses

As such, collaboration might impact two key yet understudied attitudes. First, not all healthcare workers may be absolutely convinced of the benefits of interprofessional collaboration given some of the challenges in multidisciplinary teams. Yet, belief in the benefits of interprofessional collaboration predict job satisfaction, knowledge exchange and trust [25]. Also, belief in the benefits of interprofessional collaboration is associated with patient-centered care perceptions, especially if collaboration is high. Second, interprofessional collaboration cannot be effective if team members are not able to share pertinent information [4] such as their expertise. Informational role self-efficacy is individuals’ beliefs in their capability to communicate their expertise so that it impacts others’ performance [26]. Interestingly, according to San Martin Rodriguez et al. professionals “know very little of the practices, expertise, responsibilities, skills, values and theoretical perspectives of professionals in other disciplines” [27]. It is therefore key to believe one can communicate their own expertise to others on the team, and of course engage in corresponding behaviors. Consequently, we will test the following hypothesis (see Fig. 1):

1. The relationship between (a) belief in the benefits of interprofessional collaboration and patient-centered perception and the relationship between (b) informational role self-efficacy and patient-centered perception that both occur at the individual level will be moderated by collaboration at the team level, such that both relationships will be more positive and stronger.

### Team-level perspective

Teams must adapt to face the complexities and challenges of the work they are required to accomplish [28]. As such, “adaptation lies at the heart of team effectiveness” [29]. Challenges are numerous. For example, decision-making in multidisciplinary teams is significantly more difficult than when there is less diversity around the table [30] which requires adaptation. Two intertwined sets of task-related behaviors are important for a team to adapt: adaptivity and proactivity. Team member adaptivity is the extent to which team members deal with, answer, and/or support changes that affect team roles while team member proactivity has to do with how individuals engage “in self-starting, future-directed behavior to change a team’s situation or the way the team works” [31]. Logically team member adaptivity and proactivity should lead to positive outcomes and in the case of multidisciplinary mental health teams it should impact their ability to deliver patient-centered care.

Teamwork implies task, role and resource interdependence [32] and collaboration (as defined above) is how interdependence is enacted. Maynard at al [28]. theorize that action processes mediate the relationship between task-based work and outcomes. In this study this translates into a process such as collaboration being the mechanism through which adaptivity and proactivity is transformed into patient-centered care perceptions. Consequently, we will test the following hypothesis (see Fig. 1):

2. The relationship between team adaptive and proactive behaviors and team-level patient-centered care perceptions will be mediated by collaboration.

## MethodS

### Setting

This study uses a cross-sectional multilevel multisite survey design. Mental health professionals (i.e., study participants) come from four local health care service networks in Quebec, Canada. These networks’ territory included various practice settings such as community health centers and hospitals including outpatient clinics. The territories differed based on the presence of a psychiatric hospital on the territory and whether the geographic areas were more urban or semi-urban. Populations on the four territories varied between 135,000 to 300,000. A psychiatric institute research ethics board approved the study protocol.

### Data sources and sample

Research participants were mental health professionals and managers working in public primary care or specialized mental health services, in inpatient or outpatient settings. Eligibility was based on three criteria. First, professionals had to be part of a public mental health primary care or specialized care team. Second, the team had to be composed of at least three professionals. Third, professionals had to represent at least two disciplines.

All mental health professionals and managers who met the three eligibility criteria (i.e., *N*=466) were invited to take part in a large scale study of mental health teams by way of a mailed-in questionnaire and written consent form. Those who accepted the invitation signed the consent form and filled in the questionnaire. There were two project presentations and three recruitment drives (i.e., campaigns) but no incentive to participate. Data collection spanned 20 months between October 2013 and June 2014. The 45-min questionnaire comprised 21 standardized scales and six separate questions on socio-professional characteristics adapted for mental health professionals. Of the 21 standardized scales used for the larger study, four were used in the present study (none of which required a license). A research advisory committee of 12 members, composed of representatives from the four networks, provided oversight for the study and help in gaining access to the research sites.

### Measures

#### Outcome

We adapted the Recovery Self-Assessment questionnaire [33, 34] as an outcome measure of patient-centered care perceptions. Hill et al. note “striking similarities” between recovery and patient-centeredness: both are based on a set of core values in the pursuit of health and wellness; both focus on fostering a sense of self and identity independent from that of a mental health diagnosis; both emphasize the individual’s context and the social relations within it; finally, both recovery and patient-centred approaches underscore the importance of empowering the individual [35]. The research team and a sample of representative providers reviewed the questionnaire to ensure its conformity with the Quebec health service delivery, its answerability by all types of mental health teams and conform to our definition of patient-centered care. In the end, items were rearranged in two sets answerable by providers. A first set of 8 items represent the individual level construct of patient-centered care perceptions. A sample item is “Users are encouraged to participate in program advisory boards and management meetings”. A second group of 22 items was meant to represent team-level construct of patient-centered care perceptions with items such as “Team members work hard to help users include significant others in the planning of a user’s treatment and recovery (e.g. spouses, friends, clergy, supervisor)” and “Team members encourage users to have hope and high expectations in regard to their recovery”. All items conformed with patient-centeredness as a clinical method focused on shared decision-making and empowerment of the patient [16] and assessed social functioning [36].

#### Independent variables

Two individual-level independent variables were used in this study: belief in the benefits of interprofessional collaboration and informational role self-efficacy. Belief in the benefits of interprofessional collaboration was measured using 5 items from Sicotte, D’Amour and Moreault [37] using a 7-point agree-disagree scale ¨(1-completely disagree; 7-completely agree). An example of an item is “I believe that interdisciplinary collaboration within teams allows to better meet the needs of the customer or user”. Informational role self-efficacy was measured using Chiocchio et al.’s [26] 5-item questionnaire. Participants were asked to answer items representing activities such as “Show the contribution of my area of expertise when the team needs to solve a problem.” by assessing “how confident you are in your ability to perform these activities by associating each activity with any number between 0 % and 100 %”.

Two measures represented team-level constructs: collaboration and team adaptive and proactive performance. Collaboration was measured using the 14-item questionnaire from Chiocchio et al. [22]. Items were measured with a 7-point frequency scale (1=never; 7=always). Sample items include “In our team … we communicate our ideas to each other about the work to be done” (communication); “… we carry out our tasks at the appropriate moment (synchronicity); “… we exchange information on ‘who does what’” (explicit coordination), and “… we have an implicit understanding of the assigned tasks” (implicit coordination). Six items from the work role performance questionnaire [31] were used to measure adaptive and proactive team behaviors. Items include “I respond constructively to changes in the way my team works” (adaptivity), and “I improve the way my team does things” (proactivity). Participants rated each item on a 7-point agree-disagree scale ¨ (1-completely disagree; 7-completely agree).

### Statistical analyses

Analyses that pertain to the first hypothesis were conducted using multilevel modeling. Multilevel modeling makes it possible to treat individual-level data and team-level-data at once and is especially adapted to treat non-independent nested data [38]. Analyses for the second hypothesis were conducted using conditional process and bootstrap analysis [39]. This technique is suited to mediation tests especially when the sample is small. Data collected at the individual level but representing constructs at the team level were aggregated prior to conducting the analyses [40] based on a direct consensus model [41].

## Results

Of the 466 mental health professionals, 315 filled and sent back their questionnaire for a response rate of 68%. One individual was dropped from this study because they were the only respondent from their team – data from only one person was considered unreliable to represent the team. Our final sample was 314 individuals nested in 48 teams. Chi-square tests were calculated to see whether language interacted with sex or profession. Both results were not statistically significant. T-tests were performed to compare participants on language and no statistically significant difference not assuming equal variances were revealed. Furthermore, all measures’ internal consistencies (i.e., Cronbach’s alphas) were compared across language and no substantive differences were found. Team size was 6.54 on average and members’ tenure on teams was on average 3 years. Table 1 describes the sample in more details.

**Table 1:** Description of the sample 314 individuals nested in 48 teams

		Frequency	%	Mean	(SD)
Sex	Men	96	30.6		
	Women	218	69.4		
Language^a^	French	270	87.7		
	English	38	12.3		
Age	20–29	30	9.6	43.33	(10.49)
	30–39	101	32.2		
	40–49	82	26.1		
	50–59	80	25.5		
	60–69	21	6.7		
Profession	Doctor	14	4.5		
	Nurse	94	29.9		
	Professional	175	55.7		
	Support	31	9.9		
Tenure	Months in the profession			108.03	(7.33)
	Months in the current job/position			55.61	(5.50)
	Months part of the team			36.70	(3.13)
Type of team^b^	Primary care	16	33.3		
	Outpatient SC	25	51.2		
	Inpatient SC	7	14.6		
Team size	Number of team members			6.54	(3.13)

Notes

^a^ 6 data were missing

^b^ calculated over 48 teams

*SC*: specialized care

Table 2 shows individual-level and team-level descriptive statistics. The table highlights strong reliabilities as displayed by Cronbach’s alphas. Reliabilities are also strong at the team level but this information is insufficient when preparing for multilevel modeling analyses. Aggregating data from the level at which it was measured (i.e., at the individual level) for analyses at the team level (i.e., where the constructs sit) require a number of calculations. For example, the *r*_wg(j)_ index is a measure of inter-rater agreement and the closest to + 1 the better [42]. Type 1 Intra-class correlation describes the amount of variance explained by the team-level while the type 2 index is an indicator of the reliability of the mean at the team level [40, 43]. One can see that team-level patient-centered care perceptions has 13% of variance available to be explained at the team level. Reliabilities of the means vary from .35 to .57 which is adequate given they are calculated on 48 teams. Zero-order correlations are small to moderate at the individual level and moderate to strong at the team level. Overall, these results indicate that we can proceed with analyses conducted simultaneously at the team- and individual-levels (Hypothesis 1) and with analyses conducted at the team level (Hypothesis 2).

**Table 2:** Descriptive statistics, reliability and zero-order correlations

Individual-level (*N*=314)								
	*M*	*SD*	α	1	2			
1. Belief benefits of interprof. Coll.	6.24	.73	.92					
2. Informational role self-efficacy	81.06	14.41	.93	.32 ***				
3. Patient-centered care perceptions (I)	4.16	.94	.75	.15 **	.16 **			
Team-level (*N*=48)								
	*M*	*SD*	α	*r*_wg(j)_	ICC1	ICC2	1	2
1. Team adaptive and proactive performance	5.64	.36	.86	.91	.08	.35		
2. Collaboration	4.93	.56	.94	.92	.17	.57	.63 ***	
3. Patient-centered care perceptions (T)	5.58	.44	.93	.93	.13	.49	.38 **	.47 ***

Notes

* *p* ≤ 0.05; ** *p* ≤ 0.01; *** *p* ≤ 0.001

α: Cronbach’s alpha

*r*_wg(j)_: Inter-rater agreement index with a slightly skewed null distribution (LeBreton & Senter, 2008)

ICC1: Type 1 intra-class correlation; proportion of variance accounted for by teams (Raudenbush & Bryk, 2002)

ICC2: Type 2 intra-class correlations; reliability of the team means (Bliese, 2000)

Table 3 presents results pertaining to hypotheses 1a and 1b. Multilevel modeling results show that collaboration’s main effect on individual-level patient-centered care perceptions is not statistically significant. Both individual-level variables are but belief in the benefits of interprofessional collaboration’s relationship with patient-centered care perceptions is much more substantial compared to informational role self-efficacy. The moderating effect postulated in hypothesis 1 is confirmed for belief in the benefits of interprofessional collaboration’s but not for informational role self-efficacy. Specifically, results show that the positive relationship between belief in the benefits of interprofessional collaboration and patient-centered care perceptions at the individual level is stronger when team members collaborate more intensely (hypothesis 1a). This effect was not found for hypothesis 1b and informational role self-efficacy.

**Table 3:** Parameter estimates for multilevel model

Parameter	Fixed Effects Estimate	*t*	
Intercept	4.145	54.194***	
W: Collaboration	0.245	1.737	
X1: Belief benefits of interprof. Coll.	0.207	2.409*	
X2: Informational role self-efficacy	0.009	2.111*	
W * X1	0.406	2.442*	
W * X2	−0.001	−0.099	

* *p* ≤ 0.05; ** *p* ≤ 0.01; *** *p* ≤ 0.001

Table 4 displays team-level results pertaining to hypothesis 2. The total effect of work role performance is statistically significant. The direct effect is not and the indirect effect showing the mediation of collaboration in the link between work role performance and team-level patient-centered care perceptions is statistically significant. This means that collaboration fully mediates the relationship. This result supports hypothesis 2.

**Table 4:** Total, direct and indirect effects for Team adaptive and proactive performance, Collaboration, and Team-level patient-centered care perceptions (*N*=48)

Total effect of Team adaptive and proactive performance on Team-level patient-centered care perceptions					
	Effect	SE	*t*	LLCI	ULCI
	.4568	.1656	2.81**	.1296	.7840
Direct effect of Team adaptive and proactive performance on Team-level patient-centered care perceptions					
	Effect	SE	*t*	LLCI	ULCI
	.1801	.2003	0.90	−.2233	.5836
Indirect effect of Team adaptive and proactive performance on Team-level patient-centered care perceptions					
Collaboration	Effect	Boot SE	*Z*	Boot LLCI	Boot ULCI
	.2766	.1131	2.02**	.0807	.5309

Notes

* *p* ≤ 0.05; ** *p* ≤ 0.01; *** *p* ≤ 0.001

*SE* Standard error, *LLCI* Lower level confidence interval, *ULCI* Upper level confidence interval, *Boot* Index obtained via bootstrapping

## Discussion

This study shows that it is not enough to believe in the benefits of interprofessional collaboration for these benefits to relate to individual-level patient-centered perceptions. The context of the team is central. Specifically, how team members collaborate – that is, how they communicate, coordinate, and synchronize each other – magnifies the beliefs’ association with patient-centered perceptions. Moreover, collaboration is the link between adaptive and proactive behaviors and perceptions of team-level patient-centeredness. Multidisciplinary teams should know that working hard on collaboration as an answer to the complexity of patient-centered care has two correlates. First, collaboration is related to the teams’ ability to respond to its challenges. Second, it is associated with individuals’ beliefs central to the delivery of interprofessional care.

The result regarding informational role self-efficacy is puzzling especially from the perspective of work roles. A work role is “the total set of performance responsibilities associated with one’s employment” [44]. When roles are varied in a team multiple interpretation of information and broader environmental scan occur [45]. Some mental health teams may be prone to “role-blurring and role overlap” [36] within its boundaries. We also know that in the context of mental health care teams lack of confidence signals a passive role; we also know that communicating pertinent information help establish credibility and trust [4]. These elements are related to informational role self-efficacy. Informational role self-efficacy is an individual’s beliefs in their capability to communicate their expertise so that it impacts others’ performance [26]. Perhaps the result is not statistically significant because mental health care team members already know of each other’s expertise and the need to communicate it is less relevant than in other kinds of multidisciplinary teams. It is also possible that role ambiguity and role overlap are challenging [46]. Another reason would be a dominance of medical components of care and treatment [20] which would not favor other professionals’ input. All these alternatives are worthy of future research.

This study has three main limitations. First, this is a cross-sectional study and causality cannot be established. Future studies should attempt a longitudinal design. Such design would also make it possible to measure antecedents to team adaptation and specific triggers and then see whether collaboration leads to measures of patient-centeredness. The second limitation is that the outcome measure is based on team members’ perceptions. The next step would be to measure actual patients’ perceptions. Future studies should keep the focus on multilevel modeling in order to capture team- and individual-level phenomena simultaneously. Third, there were three types of teams in this study: primary care, outpatient specialized care and inpatient specialized care. Unfortunately, the sample size (at the team level) was not large enough to compare the three kinds of teams. Team composition and professional density differ across these three settings which may have affected the results.

## Conclusions

This study showed that belief in interprofessional collaboration’s relationship with individual-level patient-centered care perceptions is increased in teams with high collaboration. We also showed that collaboration is a mediator; that is, a process by which team adaptive and proactive behaviors are transformed into positive team-level patient-centered perceptions. There was no support for informational role self-efficacy as a correlate of individual-level patient-centered care perceptions.

This study makes two contributions. First, this study establishes a team process (i.e., collaboration) as key in a multilevel examination of correlates of patient-centered care perceptions. Studies usually focus on either the team level or the individual level. And to our knowledge, studies that focus on both levels do not focus on collaboration or patient-centered care. Second, our study results are in line with recent team adaptation theory that positions communication and coordination as key mediators [28]. Our contribution was to show that collaboration is a mechanism for team adaptation as well as a context affecting beliefs about the work at hand.

## Data Availability

The datasets generated and/or analyzed during this study are not publically available because consent was not obtained for this use.

## Abbreviations

ICC1: Type 1 intra-class correlation
ICC2: Type 2 intra-class correlations
SC: Specialized care
*SE*: Standard error
LLCI: Lower level confidence interval
ULCI: Upper level confidence interval
*M*: Mean
*t*: t-test
*Z*: Z-test
